# Predictors of Incisional Hernia after Robotic Assisted Radical Prostatectomy

**DOI:** 10.1155/2015/457305

**Published:** 2015-02-02

**Authors:** Avinash Chennamsetty, Jason Hafron, Luke Edwards, Scott Pew, Behdod Poushanchi, Jay Hollander, Kim A. Killinger, Mary P. Coffey, Kenneth M. Peters

**Affiliations:** ^1^Beaumont Health System, Department of Urology, Royal Oak, MI 48073, USA; ^2^Oakland University William Beaumont School of Medicine, Rochester, MI 48309, USA; ^3^Beaumont Health System, Department of Biostatistics, Research Institute, Royal Oak, MI 48073, USA

## Abstract

*Introduction.* To explore the long term incidence and predictors of incisional hernia in patients that had RARP. *Methods.* All patients who underwent RARP between 2003 and 2012 were mailed a survey reviewing hernia type, location, and repair. *Results.* Of 577 patients, 48 (8.3%) had a hernia at an incisional site (35 men had umbilical), diagnosed at (median) 1.2 years after RARP (mean follow-up of 5.05 years). No statistically significant differences were found in preoperative diabetes, smoking, pathological stage, age, intraoperative/postoperative complications, operative time, blood loss, BMI, and drain type between patients with and without incisional hernias. Incisional hernia patients had larger median prostate weight (45 versus 38 grams; *P* = 0.001) and a higher proportion had prior laparoscopic cholecystectomy (12.5% (6/48) versus 4.6% (22/480); *P* = 0.033). Overall, 4% (23/577) of patients underwent surgical repair of 24 incisional hernias, 22 umbilical and 2 other port site hernias. *Conclusion.* Incisional hernia is a known complication of RARP and may be associated with a larger prostate weight and history of prior laparoscopic cholecystectomy. There is concern about the underreporting of incisional hernia after RARP, as it is a complication often requiring surgical revision and is of significance for patient counseling before surgery.

## 1. Introduction

Robot assisted radical prostatectomy (RARP) has emerged as the leading operation for patients with localized prostate cancer. In 2010, almost 80% of all prostatectomies in the USA were performed with robotic assistance [1]. The procedure provides patients with excellent clinical, functional, aesthetic, and oncologic results with decreased postoperative pain and quicker recovery [2]. However, in patients who develop incisional hernias, the benefits may be negated as incisional hernias can lead not only to bothersome symptoms but also to severe complications, such as bowel obstruction, strangulation, and perforation. Another reason the incidence of incisional hernias remains a concern is its reoperation rate (Grade III Clavien Classifcation); secondary repair failures as great as 45% have been reported [3, 4].

For patients who had a RARP, the incidence of incisional hernia in several large series is estimated at 0.2%–4.8% [5–13]. However, some of these studies have inadequate follow-up. Without longer follow-up, it is commonly accepted that a large number of incisional hernias are undiagnosed. In some series, 35% of all incisional hernias occurred 3 years after the operation [14]. Additionally, many asymptomatic patients may not seek medical care or present to a general surgeon directly for repair leading to a lower reported incidence. A recent review of the SEER (surveillance, epidemiology, and end results) database reported an incisional hernia repair rate after minimally invasive prostatectomy of 5.3% within 3.1 years, notably higher than previously reported in the literature [15].

Factors predisposing patients to incisional hernias include technical factors, such as trocar type and size, lack of fascial defect closure, and location of trocar placement [16]. Furthermore, the development of incisional hernias can also be affected by many predisposing host factors that decrease wound healing. When factors such as diabetes, morbid obesity, smoking, surgical site infection, malnutrition, and immunosuppression are present, optimal wound healing is impeded. The objective of our study was to explore the incidence and potential predictors of incisional hernia in patients who underwent RARP at our single teaching institution.

## 2. Materials and Methods

After receiving institutional review board approval, we identified all men who underwent RARP for prostate cancer between January 1, 2003, and December 31, 2012, by multiple surgeons at our institution. Patients of eight surgeons with varied levels of experience were included in the analysis. All patients underwent a transperitoneal approach through 6 ports using bladeless sharp or blunt obturators. Trocar placement included a 12 mm supraumbilical camera port, two 8 mm ports in the left and right lower quadrant just lateral to the rectus muscle, a 8 mm port two finger breaths above the left anterior iliac spine, a 12 mm lateral port two finger breaths above the right anterior iliac spine, and 5 mm port in the right upper quadrant. The 12 mm supraumbilical camera port site was vertically incised by all surgeons, except for one surgeon who utilized a horizontal incision. At the end of the procedure, the specimen was extracted by extending the supraumbilical site camera port site as needed. The extraction incision site was closed in an interrupted fashion with figure of eights. However suture type varied based on each individual surgeon's preference and included #0 Vicryl, #0 Prolene, #0 PDS, and #0 Ethibond. The 12 mm assistant port was routinely closed with a Carter-Thompson.

Using a mailed survey, we assessed information regarding the hernia type, location on a body diagram, and any postoperative hernia repair. Only men that answered the survey question about whether or not they had developed a hernia were included in the analysis. Hernias that occurred pre-RARP were excluded from the analysis. We also reviewed the patients' medical records for demographic, clinical, operative, and outcome characteristics. The time elapsed between date of surgery and July 15, 2013 (date that all surveys were mailed), was used to calculate the time since RARP.

Recorded data at the time of RARP included age, American Society of Anesthesiologists (ASA) score, smoking status, body mass index (BMI), operative time, intraoperative/postoperative complications, pathological stage, drain type (Jackson Pratt, penrose or none), blood loss, prostate weight, prior abdominal surgery, prior herniorrhaphy, prior laparoscopic cholecystectomy, prior appendectomy, prior colon surgery, and any other prior abdominal surgeries.

The Wilcoxon Rank Sum test, Chi-square test, and pooled *t*-tests were used to compare those who reported a postoperative incisional hernia with those who reported no hernia. Exact *P* values were obtained for the Chi-square test in the case of small expected frequencies. Multivariate logistic regression models were fit where variables were considered for inclusion if the univariate *P* values from Table 3 were ≤0.20. 95% Wald confidence intervals for odds ratios were obtained. Models with interaction terms were also considered. The fit of logistic regression models was assessed with Hosmer-Lemeshow tests and plots of model diagnostics. The SAS System for Windows version 9.3 was used for statistical analysis. Statistical significance was defined by a two-sided *P* value <0.05.

## 3. Results

We identified 1,587 patients who underwent RARP and data provided by 577 patients (36%) that returned our mailed survey were analyzed. Analysis of these 577 patients with a mean follow-up of 5.05 years revealed 48 (8.3%) patients with an incisional hernia. The hernias were diagnosed at (median) 1.2 years (IQR 0.5–2.9; range .01–9.1) after RARP.

On the body diagram, 48 patients indicated that at least one hernia was located at an incisional site, with one patient reporting two; one at an umbilical site and the other at a port site. In twelve patients that reported an incisional hernia, the actual location of the hernia was too unclear on the body diagram to report; however these were included in the total for calculation of incidence. Of the 49 total incisional hernias, 71% (35/49) were umbilical and 4% (2/49) were other ports. Both other port hernias were at the 12 mm lateral port site. Upon further review of the 35 umbilical site hernias, only one was by a surgeon who routinely used a transverse incision at the supraumbilical port site.

23 men (4%) with incisional hernias had repair of 24 incisional hernias, 22 of these men had an umbilical (extraction site) hernia repaired. The 2 hernias repaired at other sites were both the 12 mm lateral port. One patient had an incisional hernia at both sites and they were repaired simultaneously.

Of all men that returned a survey, 48/577 (8.3%) developed at least one inguinal hernia. Those patients who reported only a groin hernia and those whose reported date of hernia occurred before RARP were excluded; as a result, the number of subjects used for analysis to compare incisional hernia patients with no hernia patients was 532 (Table 1). Men with postoperative groin and inguinal hernias were included in the analysis. Of the 532 patients, the mean age was 61.5 ± 6.7 years (median 62, range 42–78), and the mean BMI was 27.80 (median 27.12, range 16.66 to 55.5). 413/525 (78.7%) had stage T2 prostate cancer. 13/532 (2.4%) had any intraoperative complications and 58/532 (11%) reported postoperative complications.

The incisional hernia (*N* = 48) patients were compared to those that did not develop a hernia (*N* = 484) on 18 potential risk factors for developing an incisional hernia after RARP. In the incisional hernia/no hernia groups, years since RARP were 4.32 ± 2.59 and 5.12 ± 2.62, respectively.

No statistically significant differences were found in preoperative diabetes, smoking, pathological stage, age, operative time, blood loss, drain type, and intraoperative and/or postoperative complications between patients with incisional hernias and those without hernias (Table 3). Of note, when we classified patients into 3 groups based on BMI (normal/underweight (BMI < 25 kg/m^2^), overweight (25 kg/m^2^ ≤ BMI < 30 kg/m^2^), and obese (BMI ≥ 30 kg/m^2^)), there was a lower rate of incisional hernia in the normal/underweight (4.7%) group while the hernia rates in the overweight (10.6%) and obese (10.1%) groups were similar (*P* = 0.15) (Table 2). Although the rate of incisional hernia was lower in normal/underweight men, the sample size was not powered to show statistical significance.

Interestingly, 17% (8/48) in the incisional hernia group and 10.3% (50/484) in the no hernia group had postoperative complications (*P* = 0.22, OR = 1.74, 95% CI for OR: (0.66, 4.04)). However, of those postoperative complications surgical site infection was not common as only 2 patients reported a surgical site infection in the no hernia group (2/464 = 0.4%) and none were reported in the incisional hernia group.

There were indications of increasing hernia rates with worse ASA physical status (*P* = 0.096). Additionally, median prostate weight was higher (45 versus 38 grams; *P* = 0.001) in the incisional hernia group compared to the no hernia group. Even though there were no statistically significant differences in prior abdominal surgery, herniorrhaphy, appendectomy, colon surgery, or any other prior abdominal surgeries between the two groups, a higher proportion of incisional hernia patients had a history of laparoscopic cholecystectomy when compared to the no hernia group (12.5% (6/48) versus 4.6% (22/480); *P* = 0.033, OR = 2.97, 95% CI for OR: (0.93, 8.10)).

Four variables were considered for inclusion in logistic regression models based on univariate *P* values and the absence of zero frequencies, which precluded fitting the model. BMI was modeled as categories (underweight/normal, overweight, and obese) rather than continuous, given the lack of fit indicated with the Hosmer-Lemeshow test. For ASA class, patients with unknown status were excluded and ASA was modeled as a continuous predictor. Our chosen model includes effects of prostate weight and whether or not the patient had a prior cholecystectomy. Larger prostate weight and history of prior cholecystectomy were associated with higher proportions of incisional hernia (Table 4). The estimated effects of these variables as measured by odds ratios are similar in the univariate logistic regressions for each variable separately, a multivariate logistic regression with four variables (Model 1: prostate weight, prior cholecystectomy, ASA, and BMI category), and a logistic regression with just prostate weight and prior cholecystectomy status (Model 2); these odds ratios are displayed in Table 4. Adjusting for prostate weight, the odds of incisional hernia for men with prior cholecystectomy are estimated to be 3.1 times the odds for men without prior cholecystectomy. Adjusting for prior cholecystectomy status, for every increase in prostate weight of five grams, the odds of incisional hernia increase by 13 percent.

## 4. Discussion

As one of the mainstays of treatment for clinically localized prostate cancer, complications of the RARP such as urinary incontinence and erectile dysfunction have been well described. However, an often overlooked complication that can contribute significantly to morbidity when incurred is incisional hernias. The cumulative incidence of incisional hernia in large open abdominal surgery ranges from 9% to 19% [3, 14, 17, 18].

This analysis of more than 550 RARP patients operated on by various surgeons revealed a relatively high incidence of incisional hernias at 8.3%. These results do not compare favorably with previously reported results in several large series ranging from 0.2% to 4.8% [5–13]. However, as the incidence of incisional hernias increases with time, many of these series may be underreporting their true incidence due to inadequate follow-up. Blatt and colleagues [5] reported a 1.9% incisional/inguinal hernia rate at 4 months of follow-up. Furthermore, they failed to make a distinction between incisional and inguinal hernias. Menon et al. [6] followed 2,652 patients for a median of 36 months, but complications were not broken down to calculate the rate of incisional hernia. Martinez-Pineiro and colleagues [7] reported a 3% incisional hernia rate in 600 patients but failed to define the time of follow-up. Similarly, Chiong et al. [8] reported a 0.9% incisional hernia rate in 441 patients without identifying the follow-up time. Our large series of 577 RARP patients with long term follow-up (mean of 5.05 years; range of 0.63–10.64 years) raises concerns about the underreporting of incisional hernia after RARP, as the higher incidence of incisional hernia is significant enough to warrant preoperative counseling.

Supporting the possibility of previously unrecognized incidence is a recent report using the SEER database that identified a 5.3% reoperative rate for incisional hernia repair after RARP at median follow-up of 3.1 years [15]. Notably, even though this rate of actual repair was higher than previously reported, the incidence of incisional hernia is likely even higher since not all incisional hernias lead to repair. The rate of reoperative repair from our study (4%) compares similarly with this large series. With a significant portion of patients requiring surgical revision of incisional hernias, urologists need to make their patients more aware of this potential complication when counseling patients for RARP.

With regard to predicted risk factors of incisional hernia such as diabetes, smoking, and BMI, statistical analysis failed to detect any significant difference between the two groups. However, when we classified patients into groups based on BMI, there was a lower rate of incisional hernia in the normal/underweight group (4.7%), whereas the hernia rates in the overweight (10.6%) and obese (10.1%) groups were higher. This can be interpreted as normal weight and is a protective factor compared to being overweight and obese. This study had an inadequate sample size precluding definitive demonstration of being overweight or obese as a significant risk factor for incisional hernia. Nonetheless, obesity remains a recognized risk factor for incisional hernia, as previous studies have identified it as an independent risk factor for incisional hernia formation [13, 19, 20]. Obese patients increased incisional hernia risk is attributed to the difficulty in fascial closure and elevated intra-abdominal pressure [21]. Moreover, these patients are at a higher risk of wound dehiscence predisposing to incisional hernia formation [22]. Additionally, there was a trend towards increasing hernia rates with worsening ASA physical status, albeit not statistically significant.

When comparing prostate weights in those with an incisional hernia and those without, we did find that men with incisional hernias had statistically significant larger prostates (medians 45 versus 38 grams; *P* = 0.001). As the umbilical incision is often extended to facilitate extraction of the specimen, prostate weight is likely a marker for incision size and particular care should be exercised in closing the fascia in patients with larger specimens. When necessary, the skin incision should likewise be extended to allow appropriate fascial closure.

Additionally, a higher proportion of incisional hernia patients had a history of laparoscopic cholecystectomy when compared to the no hernia group. It is interesting and worth noting that these two procedures share a common specimen extraction site, namely, the umbilical port site. In patients who have undergone previous laparoscopic cholecystectomy, repeat specimen extraction at the same site may contribute to weakening of the periumbilical fascia. This is indirectly supported elsewhere as incisional hernias have been reported to occur more commonly with increasing port size and in cases that use the port site for tissue extraction as in RARP [10]. It is reasonable to consider the potentially deleterious effect that repeat extractions at the same site may have on fascial integrity, and this appears to be an area in need of further investigation.

Regarding surgical technique, all patients in our series had closure of the supraumbilical incision in an interrupted fashion. Yet there is debate in minimally invasive surgery, as some recommend closing the supraumbilical incision in an interrupted suturing technique [11], while others favor the continuous approach [23]. A superior approach between these two has not been clearly elucidated with regards to limiting subsequent hernia formation.

At our institution trocar placement for a RARP traditionally includes a 5 mm port, three 8 mm ports, and another 12 mm lateral port. Both port site hernias in our series involved the 12 mm lateral port sites. The current results are consistent with a large systematic review of 19 studies, which showed that the incidence of trocar site hernias ranged from 0 to 5.2%, with an overall estimated incidence of 0.5% [24]. Review of the literature on laparoscopic surgery shows some authors recommending that all 12 mm port sites be closed [8, 25, 26]. However, controversy exists as others suggest that bladeless trocars, including the 12 mm ports, do not necessitate routine fascial closure. Rubenstein and colleagues reported no incisional hernias when they did not close the fascia of 12 mm ports in 112 port sites [27]. Additionally, Kang et al. reported no lateral 12 mm port site hernia in 498 patients where only the fascia of 12 mm midline port site was closed [10]. Despite these findings, closure of all 12 mm ports in our institution is routine.

Although our study is one of the largest reviews of postoperative incisional hernias in RARP patients with a mean follow-up of 5 years, it has limitations. Self-report of hernias is subject to error especially given the time frame between the surgery and survey completion. Survey questions may have been misinterpreted by the patient and limitations such as missing data are inherent with medical record review. Additionally, we had a relatively low questionnaire response rate of 36%, the effect of which is difficult to determine. With our high incisional hernia rate, it is possible that symptomatic patients were more apt to participate in our survey, or inversely that asymptomatic patients remain further undiagnosed. Regrettably we were unable to compare patient characteristics between responders and nonresponders and the possibility of selection bias cannot be excluded. Notwithstanding, those responding do represent a fairly sizeable number of patients and the data generated does point to both specimen size and prior cholecystectomy as significant risk factors for umbilical hernia formation, for which significance remained on multivariate analysis.

With regard to predisposing factors, we were unable to evaluate all technical factors, such as entry techniques, trocar design, and suture. All but one surgeon in our institution used a vertical incision at the supraumbilical camera port site. In an effort to reduce the rate of incisional hernias at the umbilicus, Beck and colleagues recently noted a significant reduction in midline camera port incisional hernias with the use of a transverse incision (0.6%) over a vertical incision (5.3%) [13]. The type of suture used to close the supraumbilical incision varied by surgeon and included nonabsorbable as well as absorbable suture. A large meta-analysis of randomized controlled trials of abdominal fascial closures showed level 1 evidence that there is a lower rate of incisional hernia when using a nonabsorbable suture [23]. Incisions were all closed with interrupted suture however. Our inability to evaluate proposed risk factors such as prior laparoscopic cholecystectomy and larger median prostate independent of other potential risk factors such as supraumbilical incision orientation and type of suture is a limitation of the study.

## 5. Conclusion

This study raises concerns about underreporting of incisional hernia after RARP. It is a complication often requiring surgical revision, and as such merits inclusion in preoperative counseling. Umbilical extraction site is not only where incisional hernias most likely occur but also where the vast majority of hernias significant enough to warrant repair are located. Factors such as larger prostate weight and previous laparoscopic procedures such as cholecystectomy directly affect the umbilical extraction site and may predispose to incisional hernia at this location.

## Figures and Tables

**Table 1:** Patient characteristics and surgical data for 532 patients with no hernia or incisional hernia.

	*N*	Mean ± SD (range)
Age (years)	528	61.5 ± 6.7 (42–78)
BMI		
Median (range)	530	27.2 (16.7–55.5)
Years since RARP	531	5.05 ± 2.62 (0.63–10.64)

		*N* (%)

ASA classification	513	
1		24 (4.7)
2		382 (74.5)
3		102 (20.0)
4		2 (0.4)
Prior herniorrhaphy	528	100 (18.9)
Intraoperative complication	531	13 (2.4)
Hemorrhage		1
Bladder injury		9
Epigastric artery laceration		1
Robot malfunction		2
Postoperative complication	532	58 (11)
(Grade I or II Clavien classification)		
Pathological stage	525	
T2		413 (79)
T3		112 (21)

**Table 2:** Patients classified by BMI and incisional hernia status.

	Incisional hernia	No hernia	Total
	*N* (%)	*N* (%)	
Normal/underweight (BMI < 25 kg/m^2^)	6 (4.7%)	121 (95.3%)	127 (24.0%)
Overweight (25 kg/m^2^ ≤ BMI < 30 kg/m^2^)	28 (10.6%)	237 (89.4%)	265 (50%)
Obese (BMI ≥ 30 kg/m^2^)	14 (10.1%)	124 (89.9%)	138 (26.0%)

Total	48 (9.1%)	482 (90.9%)	530

**Table 3:** Comparison of incisional hernia versus no hernia groups on potential risk factors.

	Incisional hernia	No hernia	*P* value
	*N* (%)	*N* (%)	
Diabetes	4/48 (8.3)	55/478 (11.5)	0.64
Past or current smoking	24/48 (50.0)	219/476 (46.0)	0.65
Prior abdominal surgery	18/48 (37.5)	153/479 (31.9)	0.33
Prior herniorrhaphy	9/48 (18.8)	91/480 (19.0)	1.00
Prior cholecystectomy	6/48 (12.5)	22/480 (4.6)	0.033
ASA classification	*N* = 48	*N* = 462	
1	0 (0.0)	24 (5.2)	0.096
2	35 (72.9)	347 (75.1)	
3	13 (27.1)	89 (19.3)	
4	0 (0.0)	2 (0.4)	
Intraoperative complication	0/48 (0.0)	13/483 (2.7)	0.14
Drain type	*N* = 46	*N* = 470	
None	2 (4.4)	16 (3.4)	0.68
JP	35 (76.1)	338 (71.9)	
Penrose	9 (19.6)	116 (24.7)	
Postoperative complication	8/48 (16.7)	50/484 (10.3)	0.22
(Grade I or II Clavien classification)			
Pathological stage			
T2	38/48 (79.2)	375/477 (78.6)	1.00
T3	10/48 (20.8)	102/477 (21.4)	
Age (years)			
Mean ± SD	62.46 ± 6.93	61.38 ± 6.69	0.29
Operative time (hours)			
Median (range)	3.23 (1.88–4.72)	3.27 (1.6–9.22)	0.30
Blood loss (mL)			
Median (range)	100 (10–500)	100 (10–1200)	0.60
BMI			
Median (range)	27.15 (20.7–42.9)	27.1 (16.7–55.5)	0.18
Prostate weight (grams)			
Median (range)	45.0 (17–92)	38 (13–139)	0.001

**Table 4:** Differences in incisional hernia rates as measured by odds ratios from logistic regression models.

Variable	Model 1	Model 2
	OR (95% CI)	OR (95% CI)
Prostate weight	1.13 (1.04, 1.22)	1.13 (1.05, 1.22)
(5 gm increases)		
Prior cholecystectomy	2.75 (1.03, 7.35)	3.10 (1.17, 8.20)
ASA	1.492 (0.81, 2.74)	
BMI category:		
Overweight versus normal/underweight	2.16 (0.86, 5.44)	
Obese versus overweight	0.88 (0.44, 1.78)	
Obese versus normal/underweight	1.90 (0.69, 5.21)	

Model 1 contains main effects for prostate weight, prior cholecystectomy, ASA class, and BMI category. Model 2 contains main effects for prostate weight and prior cholecystectomy.

Odds ratios for prostate weight are for a five gram increase. OR = odds ratio; 95% CI = Wald 95% CI from logistic regression.
